# Food Insecurity Is High in a Multi-Site Cohort of Transgender Women Vulnerable to or Living with HIV in the Eastern and Southern United States: Baseline Findings from the LITE Cohort

**DOI:** 10.3390/nu16050707

**Published:** 2024-02-29

**Authors:** Dougie Zubizarreta, Andrea L. Wirtz, Elizabeth Humes, Erin E. Cooney, Meg Stevenson, Keri N. Althoff, Asa E. Radix, Tonia Poteat, Chris Beyrer, Andrew J. Wawrzyniak, Kenneth H. Mayer, Sari L. Reisner

**Affiliations:** 1 Department of Social and Behavioral Sciences, Harvard T.H. Chan School of Public Health, Boston, MA 02115, USA; 2 Department of Epidemiology, Johns Hopkins Bloomberg School of Public Health, Baltimore, MD 21205, USA; awirtz1@jhu.edu (A.L.W.); ehumes1@jhu.edu (E.H.); megstevenson@jhmi.edu (M.S.); kalthoff@jhu.edu (K.N.A.); 3 Department of International Health, Johns Hopkins Bloomberg School of Public Health, Baltimore, MD 21205, USA; ecooney2@jhmi.edu; 4 Callen-Lorde Community Health Center, New York, NY 10011, USA; aradix@callen-lorde.org; 5 Department of Epidemiology, Mailman School of Public Health, Columbia University, New York, NY 10032, USA; 6 Division of Healthcare in Adult Populations, School of Nursing, Duke University, Durham, NC 27710, USA; tonia.poteat@duke.edu; 7 Duke Global Health Institute, Duke University, Durham, NC 27708, USA; chris.beyrer@duke.edu; 8 Department of Psychiatry and Behavioral Sciences, Miller School of Medicine, University of Miami, Miami, FL 33136, USA; awawrzyniak@miami.edu; 9 The Fenway Institute, Fenway Health, Boston, MA 02215, USA; kmayer@fenwayhealth.org (K.H.M.); sreisner@umich.edu (S.L.R.); 10 Department of Epidemiology, Harvard T.H. Chan School of Public Health, Boston, MA 02115, USA; 11 Department of Epidemiology, School of Public Health, University of Michigan, Ann Arbor, MI 48109, USA

**Keywords:** HIV, transgender, food insecurity

## Abstract

The prevalence and correlates of food insecurity—the unavailability of food and limited access to it—have not been adequately considered among transgender women (TW), particularly alongside other health-related conditions burdening this population, such as HIV infection. This study examined the prevalence and correlates of food insecurity among TW. Between 2018 and 2020, 1590 TW in the Eastern and Southern U.S. completed a multi-site baseline assessment (socio-behavioral survey and HIV testing). Descriptive statistics were calculated and multivariable Poisson models with robust error variance were used to estimate prevalence ratios and 95% confidence intervals for correlates of food insecurity (dichotomized as sometimes-to-always vs. seldom-to-never running out of food). Eighteen percent of TW were living with HIV and nearly half of participants (44%) reported food insecurity. Correlates of food insecurity included being Black, multiracial, or another race/ethnicity; having < college education, low income, unstable housing, and high anticipated discrimination; and a history of sex work and sexual violence (all *p* < 0.05). Food insecurity was highly prevalent among TW. Current programs to provide food support do not adequately meet the needs of TW. HIV pr evention and care programs may benefit from addressing food insecurity.

## 1. Introduction

Food insecurity—the unavailability of food and limited access to it—is a critical and understudied determinant of health that disproportionately affects socially, economically, politically, and legally marginalized women in the U.S. Transgender women face high levels of exposure to material deprivation, including poverty, homelessness, and food insecurity, which have been linked to multilevel stigma, discrimination, and violence victimization [1,2,3]. Qualitative research has found high food insecurity among transgender people in the Southeast U.S. and described barriers to seeking food assistance such as safety and fear of discrimination, as well as concerns about reducing food availability for people in greater need [4].

Transgender women are also disproportionately burdened by HIV [5,6,7]. Prior research has demonstrated that socio-structural factors, such as food insecurity, are associated with HIV and worse clinical outcomes among people living with HIV, including reduced antiretroviral therapy (ART) adherence and viral suppression, which can also lead to increased transmission risk [8,9]. Qualitative research has elucidated some potential mechanisms through which food insecurity may contribute to poor HIV outcomes for people living with HIV, including exacerbated ART side effects in the absence of food, physical feelings of hunger and fatigue, and HIV stigma at public free-meal sites [10]. While HIV research has begun to articulate the socio-structural factors driving the HIV epidemic for transgender women, the prevalence and correlates of food insecurity and its association with HIV have not been adequately considered in this population [11].

The objective of this study was to assess the prevalence and correlates of food insecurity among transgender women in the Eastern and Southern U.S. This assessment represents a critical step toward informing interventions and advancing health equity in this population.

## 2. Materials and Methods

The American Cohort to Study HIV Acquisition (known as LITE) is a multi-site longitudinal cohort study examining HIV acquisition and related health outcomes among adult transgender women in the Eastern and Southern U.S. Participants were enrolled in-person (n = 1025) at 6 U.S. cities (Atlanta, Baltimore, Boston, Miami, New York, Washington DC) or online (n = 565) from 72 cities in the same region. All participants completed a socio-behavioral survey and HIV screening.

The baseline assessment was open to all adult transgender women, regardless of HIV status. Those confirmed to be living without HIV were enrolled in the cohort; those who were living with HIV at baseline did not enroll in the cohort. All participants who screened positive for HIV antibodies at the baseline assessment were referred for confirmatory testing and care. A detailed description of recruitment and methods can be found in the published protocol [12,13].

The present study is a secondary analysis that used cross-sectional data from 1590 participants living with or without HIV who completed baseline assessments between 2018 and 2020. The Johns Hopkins School of Medicine Institutional Review Board reviewed and approved this study.

### 2.1. Measures

*Food insecurity:* Consistent with the U.S. Department of Agriculture definition of food insecurity, participants were asked, “how often do you run out of food or money to purchase food at the end of the month?” with 5-point Likert response options from “never” to “almost always” [14]. Responses were dichotomized as “sometimes to always” vs. “seldom to never”.

*Covariates:* Demographic variables included the cohort mode (online or site-based), age group in years, race and ethnicity, gender identity, U.S. census region, and year of enrollment. Socioeconomic factors were educational attainment, household income, receipt of food stamps, homelessness in the last 3 months, sex work in the last 3 months, and health insurance status. Gender affirmation factors were hormone use, surgery, and silicone use. Violence and discrimination factors included lifetime physical intimate partner violence (IPV), lifetime sexual IPV, lifetime other (non-IPV) violence, and anticipated discrimination. Baseline HIV status (laboratory-confirmed) was assessed at the baseline visit via oral specimen testing (online cohort) or the OraSure HIV self-test (site-based cohort).

### 2.2. Statistical Analysis

Missingness ranged from 0.1% to 14.6% depending on the variable. Missing data on food insecurity and the covariates were accounted for using multiple imputation by chained equations utilizing the mice package in R, version 4.2.2, to create 20 imputed datasets with 5 iterations [15]. All subsequent analyses were conducted in R on multiply imputed datasets, with estimates pooled according to Rubin’s rules [16].

Descriptive statistics (means of frequencies and proportions for 20 imputations) were calculated for the full sample and then stratified by food insecurity status. Bivariate comparisons were performed using chi-squared tests. Variables statistically significant at the alpha 0.05 level in bivariate comparisons (chi-squared tests) were included in multivariable Poisson models with robust error variance for food insecurity (Table 1). Multivariable models estimated prevalence ratios (PRs) and 95% confidence intervals (95% CIs).

## 3. Results

Table 1 presents descriptive statistics overall (N = 1590) and stratified by food insecurity status. Half of the participants were 18–29 years-old, 55% were racial/ethnic minorities, 45% had a household income ≤100% FPL, and 18% were confirmed to be living with HIV.

Food insecurity was reported by nearly half of participants (44%) and higher among people living with HIV (64%) versus those who were not (40%). Correlates of food insecurity (Table 1) included being Black (PR = 1.43; 95% CI = 1.20–1.70), multiracial, or another race/ethnicity (PR = 1.35; 95% CI = 1.15–1.58); having less than a college education [≤high school diploma/GED (PR = 1.33; 95% CI = 1.11–1.60), post-secondary education (PR = 1.28; 95% CI = 1.07–1.53)], low household income [≤100% FPL (PR = 2.08; 95% CI = 1.69–2.56), 101–200% FPL (PR = 2.03; 95% CI = 1.64–2.52)], unstable housing in the last 3 months (PR = 1.41; 95% CI = 1.27–1.57), and high anticipated discrimination [Q4 (PR = 1.44; 95% CI = 1.24–1.68)]; engaging in sex work in the last 3 months (PR = 1.21; 95% CI = 1.08–1.35); and experiencing lifetime sexual IPV (PR = 1.21; 95% CI = 1.06–1.37).

## 4. Discussion

In a sample of 1590 transgender women in the Eastern and Southern U.S., food insecurity affected more than 4 in 10 participants. Though not directly comparable due to methodology differences, the prevalence from this study far exceeds the 10.2% prevalence of food insecurity estimated among households in the U.S. population in 2021 [17]. Further, the estimate from this study was derived from data collected predominantly prior to the onset of the COVID-19 pandemic and may have worsened due to the impacts of social restrictions to prevent SARS-CoV-2 transmission [18]. Consistent with other studies, we found that socio-structural factors such as educational attainment, income, and homelessness were independently associated with food insecurity. The association with anticipated discrimination, coupled with the finding that more than half of participants who were food-insecure had post-secondary education or higher, underscores the impact of historical and ongoing discrimination faced by transgender women in the U.S. Moreover, the observed association between being Black, multiracial, or another race/ethnicity and food insecurity suggests that transgender women of color were disproportionately burdened by food insecurity, and this may be related to the compounding impact of interpersonal and structural racism and cisgenderism [19,20,21,22]. Prior research has documented associations between food insecurity and adverse health-related outcomes, including mental health conditions, violence victimization, and HIV-related outcomes [4,11,23]. To improve the health of transgender women and advance health equity, programs and interventions designed to increase access to food and address root causes of material deprivation, such as stigma and economic marginalization in this population, are needed.

Food insecurity was common among more than 60% of transgender women living with HIV, suggesting that there may be shared socio-structural drivers of food insecurity and HIV acquisition. Future intervention-actionable research examining the drivers of food insecurity in this population is needed. Moreover, the high burden of food insecurity among transgender women living with HIV suggests that programs for people living with HIV, such as the Ryan White HIV/AIDS Program that provides food and nutrition services amongst other wrap-around services, are not adequately meeting the needs of transgender women. Further, we found that there was no difference in food security across participants who did and did not receive food stamps, which suggests that receipt of food stamps alone may be insufficient for addressing the burden of food insecurity faced by transgender women. Our results highlight the need to develop additional programs and interventions to address the high burden of food insecurity faced by transgender women, regardless of HIV status, in alignment with the U.S. National HIV/AIDS strategy [24]. In addition, future qualitative work should identify barriers to participation in existing food assistance programs and develop community-informed solutions to address these barriers (e.g., peer navigator programs).

Our findings are subject to several limitations. First, the sample was restricted to transgender women living in the Eastern and Southern U.S.; thus, results may not be generalizable to transgender women living in other socio-political–legal contexts. Second, it is possible that there was misclassification bias for the outcome variable as it was measured via self-report. Third, the cross-sectional study design precludes establishing temporality and inferring causality.

## 5. Conclusions

Addressing food insecurity and other forms of material deprivation as well as mitigating barriers to food access among transgender women is a critical step in advancing health equity and changing the trajectory of HIV among transgender women in the U.S. Food insecurity and inadequate nutrition have been shown in multiple studies to undermine responses to ART among persons living with HIV infection, meaning that these findings among transgender women also have implications for their ability to achieve and sustain viral suppression and optimize their health and well-being [8,9,25].

## Figures and Tables

**Table 1:** Descriptive statistics and results from multivariable Poisson models with robust error variance for food insecurity among transgender women in the Eastern and Southern United States, 2018–2020 (N = 1590).

	Means and Frequencies Overall and by Food Insecurity Status ^a^			Multivariable Analyses ^b^
	Overall	Food Insecurity: Seldom to Never	Food Insecurity: Sometimes to Always	Outcome: Food Insecurity PR (95% CI)
N (%)	1590	892 (56.1)	698 (43.9)	
**Cohort mode**				
Site-based	1025 (64.5)	532 (59.6) *	493 (70.6) *	1.00 (Ref.)
Online	565 (35.5)	360 (40.4) *	205 (29.4) *	1.02 (0.87, 1.20)
**Age group in years**				
18–29	798 (50.2)	442 (49.6)	356 (51.0)	-
30–39	419 (26.4)	234 (26.2)	185 (26.5)	-
40+	373 (23.5)	216 (24.2)	157 (22.5)	-
**Race/ethnicity**				
White	718 (45.2)	501 (56.2) *	217 (31.1) *	1.00 (Ref.)
Black	342 (21.5)	131 (14.7) *	211 (30.2) *	1.43 (1.20, 1.70) *
Latine	303 (19.1)	156 (17.5) *	147 (21.1) *	1.18 (0.99, 1.41)
Multiracial or another race/ethnicity	227 (14.3)	104 (11.7) *	123 (17.6) *	1.35 (1.15, 1.58) *
**Educational attainment**				
High school diploma/GED or less	547 (34.4)	230 (25.8) *	317 (45.4) *	1.33 (1.11, 1.60) *
Post-secondary education	636 (40.0)	358 (40.1) *	278 (39.8) *	1.28 (1.07, 1.53) *
College graduate or more	407 (25.6)	304 (34.1) *	103 (14.8) *	1.00 (Ref.)
**Household income**				
≤100% federal poverty level	707 (44.5)	279 (31.3) *	428 (61.3) *	2.08 (1.69, 2.56) *
101–200%	323 (20.3)	161 (18.0) *	162 (23.2) *	2.03 (1.64, 2.52) *
>200%	560 (35.2)	452 (50.7) *	108 (15.5) *	1.00 (Ref.)
**Gender identity**				
Female	467 (29.4)	247 (27.7)	220 (31.5)	-
Trans woman/Transfeminine	938 (59.0)	540 (60.5)	398 (57.0)	-
Nonbinary/Another gender identity	185 (11.6)	105 (11.8)	80 (11.5)	-
**Hormone use = Yes**	1317 (82.8)	738 (82.7)	579 (83.0)	-
**Surgery = Yes**	736 (46.3)	455 (51.0) *	281 (40.3) *	0.91 (0.82, 1.02)
**Silicone = Yes**	176 (11.1)	84 (9.4)	92 (13.2)	-
**Physical IPV = Yes**	460 (28.9)	200 (22.4) *	260 (37.2) *	1.06 (0.94, 1.20)
**Sexual IPV = Yes**	443 (27.9)	188 (21.1) *	255 (36.5) *	1.21 (1.06, 1.37) *
**Other violence = Yes**	1326 (83.4)	734 (82.3)	592 (84.8)	-
**Receipt of food stamps = Yes**	492 (30.9)	185 (20.7) *	307 (44.0) *	1.12 (0.99, 1.26)
**Baseline HIV serostatus = Positive**	279 (17.5)	101 (11.3) *	178 (25.5) *	1.07 (0.94, 1.22)
**Homeless in last 3 months = Yes**	189 (11.9)	39 (4.4) *	150 (21.5) *	1.41 (1.27, 1.57) *
**Sex work in last 3 months = Yes**	265 (16.7)	76 (8.5) *	189 (27.1) *	1.21 (1.08, 1.35) *
**Uninsured = Yes**	149 (9.4)	68 (7.6) *	81 (11.6) *	1.13 (0.96, 1.32)
**Anticipated discrimination score (quartile)**				
Q1	380 (23.9)	224 (25.1) *	156 (22.3) *	1.00 (Ref.)
Q2	389 (24.5)	235 (26.3) *	154 (22.1) *	1.10 (0.94, 1.29)
Q3	415 (26.1)	252 (28.3) *	163 (23.4) *	1.16 (0.98, 1.36)
Q4	406 (25.5)	181 (20.3) *	225 (32.2) *	1.44 (1.24, 1.68) *
**Census region**				
Northeast	628 (39.5)	361 (40.5) *	267 (38.3) *	1.00 (Ref.)
Midwest	145 (9.1)	93 (10.4) *	52 (7.4) *	0.94 (0.74, 1.20)
South	817 (51.4)	438 (49.1) *	379 (54.3) *	0.91 (0.81, 1.02)
**Year of enrollment**				
2018	629 (39.6)	318 (35.7) *	311 (44.6) *	1.00 (Ref.)
2019	901 (56.7)	529 (59.3) *	372 (53.3) *	1.02 (0.91, 1.15)
2020	60 (3.8)	45 (5.0) *	15 (2.1) *	0.69 (0.44, 1.08)

Abbreviations: PR, prevalence ratio; CI, confidence interval; Ref, reference group. ^a^ Means of frequencies and percentages are calculated for 20 imputations. ^b^ PRs and 95% CIs were estimated using Poisson models with robust error variance. Estimates were pooled across 20 multiply imputed datasets. * Indicates *p*-value < 0.05 for bivariate comparisons (chi-squared tests) and indicates prevalence ratio with a corresponding 95% confidence interval that excludes the null value (i.e., 1.00) for multivariable analyses.

## Data Availability

The data presented in this study are available on request from the corresponding author.
